# Optimization of Fermentation Conditions of *Artemisia capillaris* for Enhanced Acetylcholinesterase and Butyrylcholinesterase

**DOI:** 10.3390/foods11152268

**Published:** 2022-07-29

**Authors:** Jina Choi, Jiwon Yoon, Misook Kim

**Affiliations:** Department of Food Science and Nutrition, Dankook University, Cheonan 31116, Korea; chlwlsdk96@naver.com (J.C.); wldnjs_12@naver.com (J.Y.)

**Keywords:** fermentation, *Artemisia capillaris*, optimization, Alzheimer’s disease

## Abstract

In this study, the fermentation of *Artemisia capillaris* by probiotic *Leuconostoc mesenteroides* MKJW (MKJW) was optimized to increase the acetylcholinesterase (AChE) and butyrylcholinesterase (BuChE) inhibitory and antioxidant activities using the response surface method (RSM). The independent variables were the contents of *A. capillaris*, *Gryllus bimaculatus*, and yeast extract, while the dependent variables were AChE inhibitory activity, BuChE inhibitory activity, and antioxidant activities such as FRAP, reducing power, and DPPH radical scavenging ability. Seventeen experimental runs were designed with RSM and analyzed after fermentation with MKJW. Quadratic models were used to analyze the inhibition of AChE and BuChE, and a linear model was used to analyze the FRAP. The three models were significantly appropriate (*p* < 0.0001). The highest optimal condition of the AChE inhibitory activity was derived by a multiple regression equation. When the optimum fermentation conditions were *A. capillaris* 6.75%, *G. bimaculatus* 0.18%, and yeast extract 1.27%, 91.1% was reached for AChE inhibitory, 74.0% for BuChE inhibitory, and 34.1 mM FeSO4 for FRAP. The predicted dependent variables were not significantly different from the experimental values (*p* > 0.05). In conclusion, the *A. capillaris* fermented by MKJW might be used as a natural antidementia improving agent with AChE inhibitory, BuChE inhibitory, and antioxidant activities.

## 1. Introduction

Neurological disorders are the leading causes of disability-adjusted life years (DALYs; the sum of years of life lost) and death, including chronic degenerative diseases, cancer, diabetes, and cardiovascular diseases [1,2]. The neurotransmitter acetylcholine is reduced in the brains of dementia patients compared to that in normal brains due to a decrease in acetylcholine synthesis and the breakdown of acetylcholine by cholinergic enzymes [3]. Symptoms of dementia include a decrease in memory and cognitive ability, among which Alzheimer’s disease shows decreased memory, depression, decreased judgment, and confusion. The main biological function of acetylcholinesterase (AChE) is to rapidly break down the neurotransmitter acetylcholine into acetic acid and choline [4]. Butyrylcholinesterase (BuChE) is also involved in neurological disorders as a surrogate hydrolase for acetylcholine [5]. Choline deficiency due to cholinergic enzymes is associated with the onset of Alzheimer’s disease, and the current drug approach to Alzheimer’s disease is based upon suppressing cholinergic enzymes [6]. However, common adverse effects of drugs include loss of appetite, gastrointestinal symptoms such as nausea, vomiting, diarrhea, and a lot of weight loss that appears when used for a long period [7,8]. Furthermore, no single-target drug has been successful in preventing dementia. Consequently, interest in natural plants or fermented natural products has emerged.

*Artemisia* sp., including *A. princeps*, *A. argyi*, *A. iwayomogi*, *A. princeps*, *A. capillaris*, etc. is a perennial herb with a wide distribution in Europe and Southeast Asia, including Korea [9]. *Artemisia* sp. has various physiological activities and is widely used for food or medicinal purposes [10]. In particular, many studies have been conducted on the extract of *Artemisia* sp., such as its antioxidant and anti-inflammatory effects and antibacterial, antiobesity, antileukemia, and anticancer properties [11,12,13,14]. In addition, the possibility of a natural antidementia treatment has been reported due to acetylcholinesterase inhibitory activity in the essential oil of *Artemisia* sp. [15,16].

Probiotics are living microbes that benefit consumer health by maintaining or improving the gut microbial balance [17]. Some studies on the relationship between gut microbiota and the brain have been reported [18,19]. The gut microbiota affects cognitive functions in neurodegenerative diseases related to aging and mood disorders such as anxiety and depression. The colonization of beneficial bacteria such as probiotics in the intestine catalyzes the biosynthesis of neurotransmitters and prevents neuronal damage and death [20].

Lactic acid bacteria (LAB) fermentation is a safe and green technology to preserve foods and develop novel functional foods that can modify the function of raw materials. Especially, *Leuconostoc mesenteroides* produces mannitol, dextran, and oligosaccharides as well as common LAB metabolites such as lactic acid, acetic acid, ethanol, and carbon dioxide during the fermentation process [21,22,23,24]. *Leuconostoc mesenteroides* MKJW (MKJW) is a probiotic with high resistance to digestive juices; antibacterial activity against pathogenic bacteria such as *Staphylococcus aureus*, *Enterococcus faecalis*, and *Shigella flexneri*; and high levels of antioxidant activities, such as the DPPH radical scavenging activity, superoxide dismutase (SOD)-like activity, and reducing power [24].

In this study, response surface methodology (RSM) was used to optimize the fermentation conditions (*A. capillaris*, *Gryllus bimaculatus*, and yeast extract contents) of *A. capillaris* by probiotic MKJW. Some biological activities such as AChE and BuChE inhibition and antioxidant activity of the *A. capillaris* after fermentation were investigated.

## 2. Materials and Methods

### 2.1. Materials

Hot-water-extracted *A. capillaris* (40 °brix) was obtained from DHbio (Anseong, Korea). Briefly, *A. capillaris* was extracted with ten-fold water for 6 h at 120 °C. Then, six-fold water was added, and boiled for 3 h at 105 °C. *G. bimaculatus*, obtained from Cricket Farm Co. (Hwaseong, Korea), was hot-air-dried at 70 °C for 10 h at the farm, and was ground with a mixer and sieved with an 18 mm mesh sieve. MKJW (KCTC14459BP) was used from our laboratory stock.

### 2.2. Preliminary Experiments for Fermentation Conditions

In order to determine the range of *A. capillaris* content to be input into the RSM, fermentation patterns according to *A. capillaris* at 4%, 5%, 6%, 8%, and 10% were investigated. In addition, three types of the combination of 5% *A. capillaris* and protein supplements (first, 2% (*w*/*v*) *G. bimaculatus* [Cricket Farm, Hwaseong, Korea]; second, 2% (*w*/*v*) yeast extract [Difco, Becton Dickinson and Co., Sparks, MD, USA]; and third, 1% (*w*/*v*) *G. bimaculatus* and 1% (*w*/*v*) yeast extract) were confirmed. After adding 2.5% (*w*/*v*) glucose to each preliminary test group, the mixture was sterilized for 20 min at 121 °C. After cooling, MKJW cultured for 18 h was inoculated to 5% (*v*/*v*) of the total medium volume. Then, shaking incubation (130 rpm, 30 °C) was performed for 24 h. After fermentation, the solution was pasteurized at 60 °C for 30 min and centrifuged to separate the supernatant and the precipitate.

### 2.3. Box–Behnken Experiment Design

The Box–Behnken design (BBD) can identify the linear influence, interactions between factors, and quadratic effects, and is used for RSM purposes. Response surface analysis was performed using the Stat-Ease program (Design-Expert^®^ software, version 12, Stat-Ease, Inc., Minneapolis, MN, USA) to determine the optimal *Artemisia* fermentation conditions. The amount for *A. capillaris* was between 3% and 7% (*X*_1_), for *G. bimaculatus* was between 0% and 2% (*X*_2_), and for yeast extract, was between 0% and 2% (*X*_3_), which were set as independent variables (Table 1). As dependent variables, acetylcholinesterase and butyrylcholinesterase inhibition activities and ferric reducing antioxidant activity (FRAP) were selected.

### 2.4. Fermentation

The BBD design planned 17 experiments with five replications of the center points (Table 2). The corresponding fermentation was prepared. Briefly, different contents of *A. capillaris* (3, 5, and 7%), *G. bimaculatus* powder (0, 1, and 2%), and yeast extract (0, 1, and 2%) provided from the BBD design were added into each tube, autoclaved at 121 °C for 20 min, and cooled. All fermentation broth contained sterilized 2.5% glucose. Three-times-subcultured MKJW on MRS broth (Difco, Becton Dickinson and Co., Sparks, MD, USA) was inoculated to 5% (*v*/*v*) of the total broth volume (8 Log CFU∙g^−1^). The fermentation was carried out with shaking incubation at 130 rpm and 30 °C for 12 h.

The fermented samples were serially diluted with 0.1% peptone water and spread onto the MRS agar. MRS agar was incubated for 24 h at 30 °C to determine total viable bacterial counts. For other analysis, the sample tubes were heated at 60 °C for 30 min, followed by centrifugation to collect the supernatant for use in the experiment. The pH of the samples was measured with a pH meter (Fisher Science Education, Waltham, MA USA).

### 2.5. Acetylcholinesterase (AChE) and Butyrylcholinesterase (BuChE) Inhibitory Activity

AChE and BuChE inhibitory assays were carried out in a 96-well plate using a modified method, as described by Sharififar et al. [25]. The reagents (Sigma Aldrich, St. Louis, MO, USA) were injected into the well in the following procedure: 5 µL enzyme solution (AChE or BuChE, 2 U/mL), 40 µL 5,5-dithio-bis-(2-nitrobenzoic acid) (DTNB, 1 mM), and 125 µL diluted sample solution. The mixture was pre-incubated for 5 min at 25 °C. The reaction was initiated by adding 7.5 µL substrate (acetylcholine iodide or butyrylthiocholine iodide, 7.5 mM), and then the absorbance was measured at 412 nm for 15 min at 25 °C. The control consisted of adding the phosphate buffer (200 mM, pH 7.7) instead of the sample. All solutions were dissolved in 200 mM phosphate buffer (pH 7.7). Absorbance was plotted against time and enzyme activity was calculated from the slope of the line, obtained and expressed as a percentage compared with an assay using a buffer without inhibitor. Galantamine was used as the positive control.

% AChE and BuChE inhibitory activity = [1 − (S_sample_/S_blank_)] × 100 S_sample_: Slope of the sample S_blank_: Slope of the control (1)

### 2.6. Antioxidant Activity

#### 2.6.1. DPPH Radical Scavenging Activity

The activity to scavenge the free radical DPPH was measured according to the method of Park et al. [26], with modifications. Briefly, a volume of 300 µL of the sample solution was mixed with 200 mM 2,2-diphenyl-1-picrylhydrazyl (DPPH) solution dissolved in methanol, which was left to react for 30 min at room temperature in the dark. The amount of scavenged DPPH radical was determined by measuring the absorbance at 517 nm. Methanol was used as the control instead of the sample. Ascorbic acid was used as the positive control. The DPPH radical scavenging activity was calculated as follows:

% DPPH radical scavenging activity = [1 − (A_control_ − A_sample)_/A_control_] × 100 A_control_: Absorbance of the control A_sample_: Absorbance of the sample (2)

#### 2.6.2. Reducing Power

The method developed by Lin and Yen [27] was used with modifications to evaluate the reducing activity of fermented samples. Then, 250 µL sample was mixed with an equal volume of 200 mM sodium phosphate buffer (pH 6.6) and 1% (*w*/*v*) potassium ferricyanide solution. The reaction mixture was incubated at 50 °C for 20 min, then 250 µL of 10% (*w*/*v*) trichloroacetic acid was added to stop the reaction. Next, 500 µL the upper phase was obtained by centrifugation at 10,000 rpm for 5 min. Then, 500 µL of distilled water and 100 µL of 0.1% (*w*/*v*) FeCl_3_ was added. The absorbance was read at 700 nm. Ascorbic acid was used to the positive control. The reducing power was expressed as the absorbance value at Abs 700 nm.

#### 2.6.3. Ferric Reducing Antioxidant Power (FRAP)

The FRAP value of the fermented samples was determined using the method described by Cermeno et al. [28], with some modifications. The FRAP reagent was prepared by mixing 300 mM acetate buffer, 10 mM 2,4,6-tripyridyl-s-triazine (TPTZ), and 20 mM FeCl_3_∙6H_2_O in a 10:1:1 (*v*/*v*/*v*) ratio and heating at 37 °C for 15 min. Then, 1 mL of FRAP reagent was added to 50 µL of the sample solution. Then, the absorbance was read at 590 nm after 30 min incubation at 37 °C. The FRAP value was expressed as the millimole (mM) of the FeSO_4_ equivalents.

### 2.7. Total Polyphenol Contents

The total polyphenol content (TPC) was determined by the Folin–Ciocalteu method, according to a method adapted from Anesini et al. [29]. 0.2 mL of the sample solution was mixed with 1.0 mL of Folin–Ciocalteu reagent, which was diluted 10 times in water. Then, 0.8 mL of 7.5% (*w*/*v*) Na_2_CO_3_ was added and the mixture was left to stand at room temperature for 1 hr. The absorbance was measured at 765 nm. The TPC was expressed as the mg of gallic acid equivalents per ml of sample (mg GAE/mL).

### 2.8. Statistical Analysis

The data were expressed as the means ± standard deviation of triplicates. Analysis of variance and Tukey’s means comparison tests were performed to determine the significant differences (*p* < 0.05) in the results using Minitab 16.0 software (Minitab Inc., State College, PA, USA). The influence of the independent variable on the dependent variable was quantified by deriving a quadratic regression equation, and the optimal condition was predicted by plotting a three-dimensional response surface diagram using the Stat-Ease program (Design-Expert^®^ software, version 12, Stat-Ease, Inc., Minneapolis, MN, USA).

## 3. Results and Discussion

### 3.1. Preliminary Experiments for Fermentation Conditions

**Figure 1:**
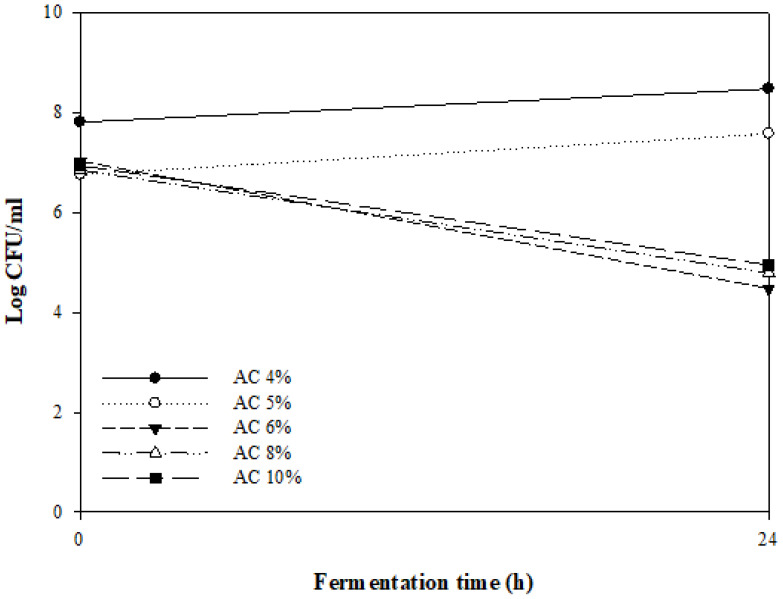
Changes in the viable cell counts of MKJW-fermented *A. capillaris* (AC) during 24 h.

Figure 1 shows the number of viable bacteria when different concentrations of *A. capillaris* (4–10%) were fermented by MKJW for 24 h. The fermentation was successfully performed when 4% and 5% *A. capillaris* were used. However, more than 5% *A. capillaris* did not proliferate. *Artemisia* sp. has been reported to inhibit the growth of even lactic acid bacteria such as *Lactobacillus plantarum* and *Leuconostoc mesenteroides*, which are commonly used as fermentation starters [30,31], as well as pathogenic bacteria such as *Bacillus subtilis* [32,33], *Escherichia coli* [32,33], *Listeria monocytogenes* [34], *Pseudomonas aeruginosa* [32], *Staphylococcus aureus* [32,33,34], and *Saccharomyces cerevisiae* [35].

**Figure 2:**
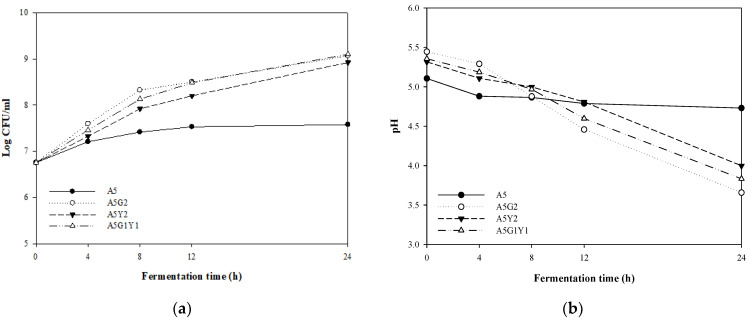
Comparison of changes in the viable cell counts (**a**) and pH (**b**) of MKJW-fermented *A. capillaris* during 24 h. A5, *A. capillaris* 5%; A5G2, *A. capillaris* 5% + *G. bimaculatus* 2%; A5Y2, *A. capillaris* 5% + yeast extract 2%; A5G1Y1, *A. capillaris* 5% + *G. bimaculatus* 1% + yeast extract 1%.

Figure 2 shows the number of viable bacteria and pH changes that occurred during 24 h with the addition of each *G. bimaculatus* and yeast extract to 5% *A. capillaris*. The addition of supplemental materials such as *G. bimaculatus* and yeast extract to *A. capillaris* enhanced the growth of MKJW to approximately 9 log CFU/mL compared to the 5% *A. capillaris* only (7.58 log CFU/mL) during 24 h fermentation. The pH of 5% *A. capillaris* decreased from 5.11 to 4.73 for 24 h; 5% *A. capillaris* supplemented with 2% *G. bimaculatus* decreased from 5.45 to 3.66 after fermentation; 5% *A. capillaris* supplemented with 2% yeast extract decreased from 5.32 to 4.00; and 5% *A. capillaris* supplemented with 1% *G. bimaculatus* and 1% yeast extract decreased from 5.36 to 3.83.

As a result, MKJW did not grow well when fermented with only *A. capillaris*, but the bacteria grew well as a result of fermentation with the addition of the protein-rich *G. bimaculatus* and yeast extract. *A. capillaris* is thought to have poor growth due to a lack of nitrogen source necessary for the synthesis of protein, enzymes, nucleic acid, and other microbial components among the nutrients that are necessary for the growth of lactic acid bacteria. Park et al. [36] fermented glasswort witha mixture of rice bran and soybeans to supplement sugar and protein. When Yang et al. [37] mixed and fermented amaranth and quinoa to compensate for the protein source lacking in white rice, the number of viable bacteria increased compared to when only white rice was fermented.

Finally, the *A. capillaris* content was set to 5%, which allowed the bacteria to grow. The fermentation time was determined to be 12 h when the bacteria grew well, and the antioxidant activity was high.

### 3.2. Experiment Design for the Optimal Condition of A. capillaris Fermentation

#### 3.2.1. Microbiological and Physiological Analysis of Fermented *A. capillaris*

Based on the preliminary experiments, the amounts of *A. capillaris*, *G. bimaculatus*, and yeast extract were selected as independent variables. Fermentation of *A. capillaris* was carried out under 17 conditions according to the Box–Behnken design. Table 2 shows the changes in the viable cell counts, pH value, and dependent variables during fermentation for the 17 experimental runs. All samples increased in viable cells and decreased in pH during fermentation. This result shows that MKJW eats nutrients such as glucose and yeast extracts, and as it grows, it produces fermentation products such as organic acids [38].

The substrate combination of 3% *A. capillaris*, 2% G. *bimaculatus*, and 1% yeast extract, had a significant increase in viable cell counts (8.99 Log CFU/mL), and a significant decrease in pH values. The fermentation with supplementation of *G. bimaculatus* or/and yeast extract to *A. capillaris* boosted the fermentation with increasing cell growth and lowering the pH values.

#### 3.2.2. Suitability of the Regression Model

The results were analyzed using multiple regression, and the coefficients of each model were evaluated for their significance by regression analysis (Table 3). The model of the AChE inhibitory activity was adopted as a quadratic model in which independent variables interact. The F-value of 32.83 implied that the model was significant. *p*-values less than 0.05 indicated that model terms (factors *X*_1_, *X*_2_, *X*_3_, *X*_1_*X*_2_, *X*_2_*X*_3_, *X*_1_^2^) were statistically significant. In addition, F-value (1.94) of the lack of fit was not significant (*p* = 0.26) relative to the pure error. A nonsignificant lack of fit is a good indication that the model fits the actual relationships of the response parameters within the chosen ranges. Likewise, the model of the BuChE inhibitory activity was adopted as a quadratic model. The F-value of 99.08 and *p*-values less than 0.05 showed that the model was significant. The factors, *X*_1_, *X*_2_, *X*_1_*X*_2_, *X*_1_*X*_3_, *X*_2_*X*_3_, *X*_1_^2^, *X*_2_^2^, and *X*_3_^2^, were highly significant. The lack of fit was also not significant (F = 1.21, *p* = 0.4129) indicating fit goodness of the proposed model [34]. The values of the F-(77.12) and *p*-(<0.0001) was to indicate that FRAP (*Y*_3_) is suitable for the linear model. Furthermore, the *p*-value for all primary terms was less than 0.05 as a result of the analysis of variance. This means that each independent variable of all linear terms affects the dependent variable. F-value of the lack of fit was 1.77 and the *p*-value was 0.31, which was more than 0.1, thus indicating that the model is appropriate. The lack of fit of the reducing power (*Y*_4_) and DPPH radical scavenging ability (*Y*_5_), which are for the remaining antioxidant experiments, were 0.0983 and 0.0742, respectively. For each, the lack of fit was lower than 0.1, so the two experiments were determined to be inappropriate models.

Table 4 shows the actual and predicted values of each dependent variable (AChE inhibitory activity, BuChE inhibitory activity, and FRAP) based on the experimental design. The observed and predicted values matched reasonably well.

#### 3.2.3. Model Verification

The final estimated response model equation for the AChE inhibitory activity was as follows:

*Y*_1_ = 74.60 + 15.13 *X*_1_ − 7.46 *X*_2_ + 5.16 *X*_3_ − 4.10 *X*_1_*X*_2_ − 0.6500 *X*_1_*X*_3_ + 4.42 *X*_2_*X*_3_ − 7.34 *X*_1_^2^ + 0.2375 *X*_2_^2^ − 2.36 *X*_3_^2^ (3)

The coefficients of *A. capillaris* (*X*_1_) and the yeast extract (*X*_3_) were positive; *G. bimaculatus* (*X*_2_) was negative of the dependent variable *Y*_1_. This means that when the contents of *A. capillaris* (*X*_1_) and yeast extract (*X*_3_) increase and the content of *G. bimaculatus* (*X*_2_) decreases, the AChE inhibitory activity (*Y*_1_) increases. The response increased as the content of yeast extract (*X*_3_) increased, but the influence on the response was less than that of *A. capillaris* (*X*_1_). The adjusted coefficient of determination for the multiple regression equation was 0.9471 and the *p*-value was less than 0.0001.

**Figure 3:**
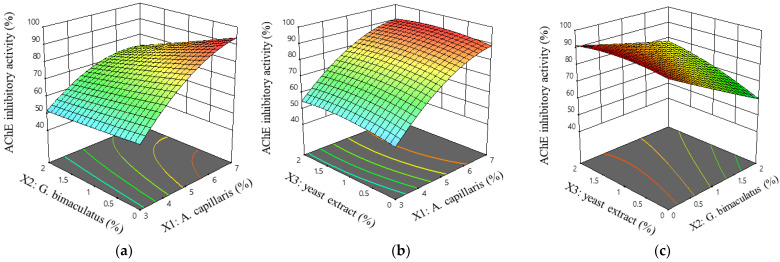
Response surface and contour plots for the AChE inhibitory activity of MKJW-fermented *A. capillaris*. *A. capillaris* and *G. bimaculatus* (**a**); *A. capillaris* and yeast extract (**b**); *G. bimaculatus* and yeast extract (**c**).

Equation (3) was visualized as a 3D response surface and is shown in Figure 3. Figure 3a shows that the AChE inhibitory activity was rapidly increasing in a curved shape when the *A. capillaris* (*X*_1_) content increased, which then decreased in response to the increase in the *G. bimaculatus* (*X*_2_) content. Figure 3b shows the effect of the *A. capillaris* (*X*_1_) content and yeast extract (*X*_3_) content response, and as in 3a, it increased as the *A. capillaris* (*X*_1_) content increased, but with little effect on the increase of yeast extract (*X*_3_) content. Figure 3c shows that the lower the content of *G. bimaculatus* (*X*_2_), the lower the change in the AChE inhibitory activity as the yeast extract (*X*_3_) increased. Conversely, as the content of *G. bimaculatus* (*X*_2_) increased, the effect of the yeast extract (*X*_3_) on the AChE inhibitory activity increased.

The coefficient for BuChE inhibitory activity (*Y*_2_) was positive for *A. capillaris* (*X*_1_) and yeast extract (*X*_3_), and negative for *G. bimaculatus* (*X*_2_). This result was the same as *Y*_1_. As the contents of *A. capillaris* (*X*_1_) and yeast extract (*X*_3_) increased and the content of *G. bimaculatus* (*X*_2_) decreased, the BuChE inhibitory activity (*Y*_2_) value increased. The adjusted coefficient of determination was 0.9822 and the *p*-value was less than 0.0001. The final estimated response model equation for BuChE inhibitory activity is as follows:

*Y*_2_ = 50.10 + 16.13 *X*_1_ − 2.96 *X*_2_ + 0.3375 *X*_3_ − 6.52 *X*_1_*X*_2_ − 6.08 *X*_1_*X*_3_ + 3.65 *X*_2_*X*_3_ + 2.87 *X*_1_^2^ + 4.15 *X*_2_^2^ − 4.80 *X*_3_^2^ (4)

**Figure 4:**
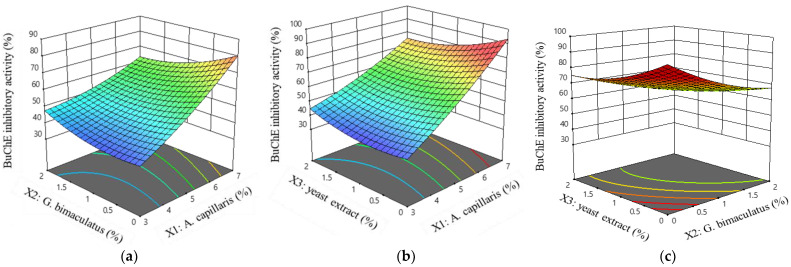
Response surface and contour plots for the BuChE inhibitory activity of MKJW-fermented *A. capillaris*. *A. capillaris* and *G. bimaculatus* (**a**); *A. capillaris* and yeast extract (**b**); *G. bimaculatus* and yeast extract (**c**).

Equation (4) was consistent with the three-dimensional graph that shows the interaction between factors, which is presented in Figure 4. Figure 4a,b show that the BuChE inhibitory activity increased rapidly as the *A. capillaris* (*X*_1_) increased. In addition, the response was decreased when the content of *G. bimaculatus* (*X*_2_) or yeast extract increased. Figure 4c shows that the response decreased as the yeast extract (*X*_3_) and *G. bimaculatus* (*X*_2_) increased.

The coefficient for FRAP (*Y*_3_) was positive for *A. capillaris* (*X*_1_) and yeast extract (*X*_3_), whereas *G. bimaculatus* (*X*_2_) was negative. *G. bimaculatus* (*X*_2_) has less influence on the dependent variable than do *X*_1_ and *X*_3_. Its R^2^ was 0.9468. The final estimated response model equation for the FRAP value is as follows:

*Y_3_* = 24.33 + 7.84 *X*_1_ − 1.68 *X*_2_ + 3.09 *X*_3_ (5)

**Figure 5:**
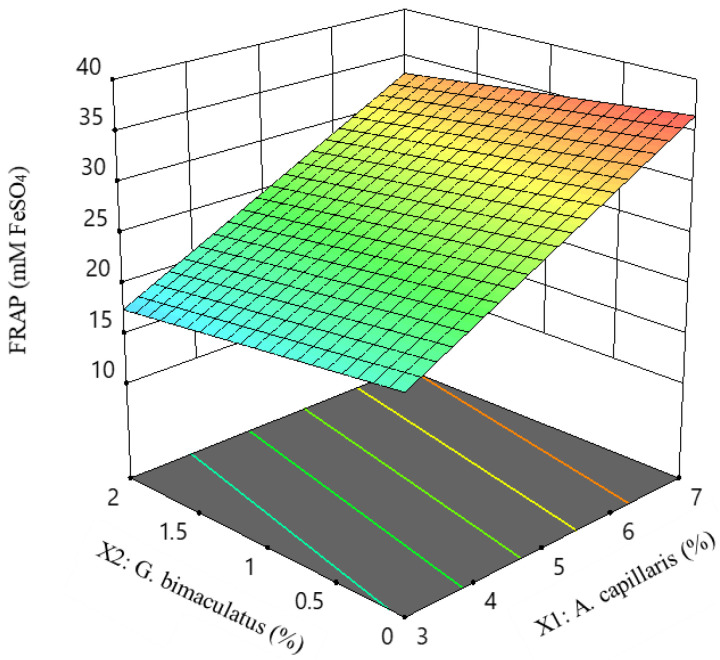
Response surface and contour plots for FRAP value of MKJW-fermented *A. capillaris*.

Figure 5 shows the changes in the independent variables *A. capillaris* (*X*_1_) and *G. bimaculatus* (*X*_2_) when the independent variable yeast extract (*X*_3_) was fixed. FRAP was only significant for linear terms (*p* < 0.05). As with the regression equation, as *A. capillaris* (*X*_1_) increased, the FRAP value increased linearly.

Based on our results, the models for *Y*_1_, *Y*_2_, and *Y*_3_ can be useful tools to optimize the fermentation conditions because these are statistically significant and provide solid solutions as a function of independent variables.

### 3.3. Optimization of A. capillaris Fermented Product

**Figure 6:**
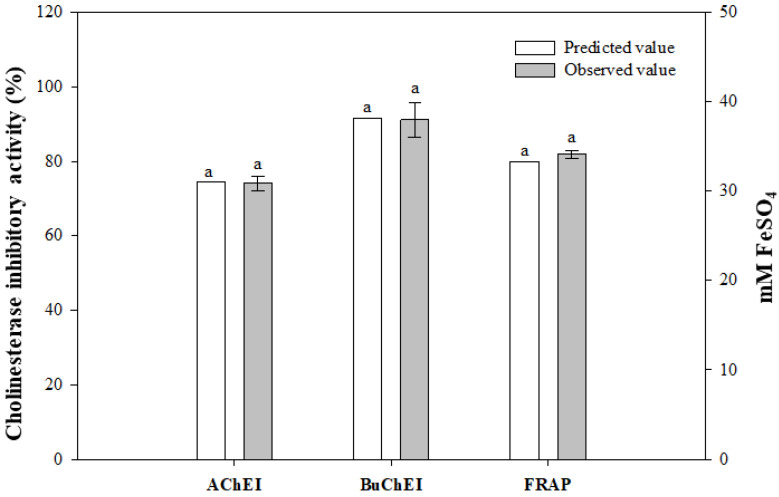
Comparison of predicted and observed values of MKJW-fermented *A. capillaris.* Same letters between the predicted and observed value are not significantly different (*p* > 0.05).

The optimal fermentation conditions were determined by maximizing the desirability of the responses. The maximal desirability should be initially at the highest values for AChE and BuChE inhibitory activities, and FRAP, and then be at the higher concentration of *A. capillaris*. Finally, the optimal fermentation conditions for AChE and BuChE inhibitory activities are as follows: *X*_1_ = 6.75%, *X*_2_ = 0.18%, and *X*_3_ = 1.27%. Inoculum volume was 6.82 increased to 8.14 with a decrease in pH values from 5.16 to 4.93. Using the above conditions, the predicted values for each model are *Y*_1_ = 91.6%, *Y*_2_ = 74.5%, and *Y*_3_ = 33.3 mM FeSO_4_. After fermentation under optimal conditions, the predicted and observed values for the three dependent variables were analyzed (Figure 6). The observed values of each dependent variable were the AChE inhibitory activity (*Y*_1_) of 91.1 ± 4.75% (*p* = 0.864), BuChE inhibitory activity (*Y*_2_) of 74.0 ± 1.96% (*p* = 0.681), and FRAP (*Y*_3_) of 34.1 ± 0.43 mM FeSO_4_, (*p* = 0.060). There was no significant difference between the predicted value and the observed value. These results reveal that the model used in this study is valid.

The antioxidant activities and TPC of fermented *A. capillaris* under optimal conditions are shown in Table 5. The DPPH radical scavenging activity was 86.5 ± 0.89%. It was higher than *Lactobacillus*-fermented *A. annua* (80%) during 12 h, which was reported by Lee et al. [39]. The reducing power was 2.14 ± 0.02 at Abs 700 nm. The total polyphenol content of fermented *A. capillaris* was 1.52 ± 0.61 mg GAE/mL. It was a much higher content compared to other fermented *Artemisia* sp. The total polyphenol contents of *A. annua* L. fermented by *Lactobacillus* sp. for 12 h were 0.3 mg GAE/mL [39], and those of *A. princeps* Pamp. fermented by *Monascus* sp. were about 0.7 mg GAE/mL [40]. This is thought to be because the *Artemisia* sp. type and starter are different. A significant correlation between total polyphenol content and AChE inhibitory activity among the five *Asteraceae* sp. plants has been reported [41]. The aromatic phenyl groups (-C_6_H_5_) of polyphenols structurally interact well with the active site of AChE, which forms as an aromatic residue to inhibit the decomposition of acetylcholine [42]. Aside from the onset of Alzheimer’s disease by cholinesterase, another risk factor for Alzheimer’s disease is that oxidative stress increases in aging patients due to the accumulation of reactive oxygen species [43]. Oxidative stress can be eliminated by antioxidant active substances such as polyphenols and exopolysaccharides. Papandreou et al. [44] reported that when polyphenol-rich blueberry extracts were administered in the mouse brain, both oxidative stress and AChE activity reduced. The antioxidant active substances can be produced or increased as a result of fermentation using lactic acid bacteria [45]. Therefore, it is believed that the antioxidant activity of the raw *A. capillaris* increased further due to the fermentation of MKJW as well as AChE inhibitory activity.

## 4. Conclusions

This study showed that optimized fermentation of *A. capillaris* by *L. mesenteroides* MKJW increased its cholinesterase inhibition activity and antioxidant activity. The maximal activity of acetylcholinesterase (91.6%) and butyrylcholinesterase (74.5%) inhibition and FRAP (33.3 mM FeSO_4_) was achieved when 6.75% *A. capillaris* was fermented by MKJW in the presence of 0.18% *G. bimaculatus* and 1.27% yeast extract. Therefore, it is thought that it can be used as probiotic *A. capillaris* possessing antidementia and antioxidant properties, but further research should be undertaken.

## Figures and Tables

**Table 1:** Levels of independent variables in the Box–Behnken experimental design.

Independent Variables	Levels		
	Low	Mid	High
*X*_1_: *A. capillaris* (%)	3	5	7
*X*_2_: *Gryllus bimaculatus* (%)	0	1	2
*X*_3_: Yeast extract (%)	0	1	2

**Table 2:** Change of the viable cell counts, pH value, and physiological activities of MKJW fermented *A. capillaris*.

Run	*A. capillaris* (%, *X*_1_)	*G. bimaculatus* (%, *X*_2_)	Yeast Extract (%, *X*_3_)	Log CFU/mL ^1^		pH		AChE Inhibitory Activity (%, *Y*_1_)	BuChE Inhibitory Activity (%, *Y*_2_)	FRAP (mM FeSO_4_, *Y*_3_)	Reducing Power (%, *Y*_4_)	DPPH ^2^ (%, *Y*_5_)
				0 h	12 h	0 h	12 h					
1	5	1	1	6.85	8.34	5.36	4.84	73.8	50.0	24.7	34.1	43.1
2	5	1	1	6.85	8.41	5.36	4.86	78.0	52.6	21.5	31.5	42.2
3	7	2	1	6.85	8.28	5.28	5.08	69.9	64.4	28.5	39.3	53.3
4	5	2	0	6.85	8.48	5.45	4.76	56.2	50.6	20.6	24.3	30.9
5	5	1	1	6.85	8.39	5.36	4.79	74.3	48.3	24.6	31.4	39.7
6	5	1	1	6.85	8.43	5.36	4.73	70.9	48.9	23.5	31.5	39.4
7	3	1	0	6.85	8.68	5.15	4.15	40.9	36.4	12.6	13.8	18.5
8	7	0	1	6.85	7.57	5.19	5.02	91.9	82.4	33.2	42.2	62.8
9	5	0	2	6.85	7.97	5.32	5.03	79.9	60.2	30.7	44.6	60.5
10	7	1	0	6.85	7.73	5.24	5.12	75.9	80.5	29.4	35.1	52.4
11	3	0	1	6.85	8.51	5.11	4.36	56.9	36.8	17.5	24.6	27.6
12	5	2	2	6.85	8.61	5.37	4.77	72.7	60.6	27.1	40.8	45.9
13	3	2	1	6.85	8.99	5.44	4.22	51.3	44.9	16.8	22.8	23.4
14	7	1	2	6.85	8.06	5.34	5.08	87.6	67.0	36.5	51.9	66.7
15	5	0	0	6.85	7.53	5.11	4.99	81.1	64.8	25.0	30.0	43.7
16	5	1	1	6.85	8.38	5.36	4.81	76.0	50.7	23.4	30.7	39.2
17	3	1	2	6.85	8.90	5.41	4.36	55.2	47.2	18.0	24.5	26.6

^1^ The viable cell number at 0 h and 12 h fermentation. ^2^ DPPH radical scavenging activity.

**Table 3:** ANOVA results for dependent variables.

	AChE Inhibitory Activity (*Y*_1_)		BuChE Inhibitory Activity (*Y*_2_)		FRAP (*Y*_3_)	
	F-Value	*p*-Value	F-Value	*p*-Value	F-Value	*p*-Value
Model ^1^	32.83	<0.0001	99.08	<0.0001	77.12	<0.0001
*X*_1_	186.78	<0.0001	674.66	<0.0001	192.66	<0.0001
*X*_2_	45.47	0.0003	22.77	0.0020	8.80	0.0109
*X*_3_	21.76	0.0023	0.2956	0.6036	29.90	0.0001
*X*_1_ *X*_2_	6.86	0.0344	55.24	0.0001		
*X*_1_ *X*_3_	0.1725	0.6904	47.88	0.0002		
*X*_2_ *X*_3_	7.99	0.0255	17.28	0.0043		
*X*_1_^2^	23.14	0.0019	11.29	0.0121		
*X*_2_^2^	0.0242	0.8807	23.52	0.0019		
*X*_3_^2^	2.40	0.1654	31.46	0.0008		
Lack of fit	1.94	0.2650	1.21	0.4129	1.77	0.3053

^1^ Model: *Y*_1_, *Y*_2_ = Quadratic, *Y*_3_ = Linear.

**Table 4:** Observed and predicted values for AChE, BuChE inhibitory activity (%), and FRAP (mM FeSO_4_) of MKJW-fermented *A. capillaris*.

Run	AChE Inhibitory Activity (%)			BuChE Inhibitory Activity (%)			FRAP (mM FeSO_4_)		
	Observed *Y*	Predicted *Y*	Residuals	Observed *Y*	Predicted *Y*	Residuals	Observed *Y*	Predicted *Y*	Residuals
1	73.80	74.60	–0.80	50.00	50.10	–0.10	24.70	24.33	0.37
2	78.00	74.60	3.40	52.60	50.10	2.50	21.50	24.33	–2.83
3	69.90	71.06	–1.16	64.40	63.76	0.64	28.50	30.49	–1.99
4	56.20	55.43	0.78	50.60	52.10	–1.50	20.60	19.57	1.03
5	74.30	74.60	–0.30	48.30	50.10	–1.80	24.60	24.33	0.27
6	70.90	74.60	–3.70	48.90	50.10	–1.20	23.50	24.33	–0.83
7	40.90	43.96	–3.06	36.40	35.24	1.16	12.60	13.40	–0.80
8	91.90	94.19	–2.29	82.40	82.74	–0.34	33.20	33.84	–0.64
9	79.90	80.68	–0.77	60.20	58.70	1.50	30.70	29.09	1.61
10	75.90	75.51	0.39	80.50	79.64	0.86	29.40	29.08	0.32
11	56.90	55.74	1.16	36.80	37.44	–0.64	17.50	18.17	–0.67
12	72.70	74.60	–1.90	60.60	60.08	0.52	27.10	25.74	1.36
13	51.30	49.01	2.29	44.90	44.56	0.34	16.80	14.82	1.98
14	87.60	84.54	3.06	67.00	68.16	–1.16	36.50	35.25	1.25
15	81.10	79.20	1.90	64.80	65.33	–0.53	25.00	22.92	2.08
16	76.00	74.60	1.40	50.70	50.10	0.60	23.40	24.33	–0.93
17	55.20	55.59	–0.39	47.20	48.06	–0.86	18.00	19.58	–1.58

**Table 5:** Antioxidant activities and TPC of fermented *A. capillaris* under optimal conditions.

	DPPH Radical Scavenging Activity (%)	Reducing Power (Abs at 700 nm)	FRAP (mM FeSO_4_)	TPC (mg GAE/mL)
Optimal conditions ^1^	86.5 ± 0.89	2.14 ± 0.02	34.1 ± 0.43	1.52 ± 0.61
Ascorbic acid ^2^	97.3 ± 0.65	2.68 ± 0.15	-	-

^1^*A. capillaris*: 6.75%, *G. bimaculatus*: 0.18%, yeast extract: 1.27%. ^2^ Ascorbic acid 1 mg/mL.

## Data Availability

The data presented in this study are available on request from the corresponding author.
